# Curcumin-Based Molecularly Imprinted Polymer Electropolymerized on Single-Use Graphite Electrode for Dipyridamole Analysis

**DOI:** 10.3390/molecules29194630

**Published:** 2024-09-29

**Authors:** Daniel Preda, Gabriel Lucian Radu, Emilia-Elena Iorgulescu, Mihaela-Carmen Cheregi, Iulia Gabriela David

**Affiliations:** 1 Doctoral School of Chemical Engineering and Biotechnologies, National University of Science and Technology Politehnica Bucharest, Gheorghe Polizu Street 1-7, District 1, 011061 Bucharest, Romania; danielpredaa12@gmail.com; 2 National Institute of Biological Sciences, Centre of Bioanalysis, Splaiul Independentei 296, District 6, 060031 Bucharest, Romania; 3 Department of Analytical Chemistry and Physical Chemistry, Faculty of Chemistry, University of Bucharest, Panduri Avenue 90-92, District 5, 050663 Bucharest, Romania; emilia-elena.iorgulescu@chimie.unibuc.ro (E.-E.I.); mihaela.cheregi@g.unibuc.ro (M.-C.C.)

**Keywords:** dipyridamole, molecularly imprinted polymer, modified electrodes, electroanalysis, pencil graphite electrode, disposable electrode

## Abstract

A new molecularly imprinted polymer (MIP)-based disposable electrochemical sensor for dipyridamole (DIP) determination was obtained. The sensor was rapidly prepared by potentiodynamic electrochemical polymerization on a pencil graphite electrode (PGE) using curcumin (CUR) as a functional monomer and DIP as a template molecule. After the optimization of the conditions (pH, monomer–template ratio, scan rate, number of cyclic voltammetric cycles applied in the electro-polymerization process and extraction time of the template molecule) for MIP formation, DIP voltammetric behavior at the modified electrode (MIP_PGE) was investigated. DIP oxidation took place in a pH-dependent, irreversible mixed diffusion-adsorption controlled process. Differential pulse voltammetry (DPV) and adsorptive stripping differential pulse voltammetry (AdSDPV) were used to quantify DIP from pharmaceutical and tap water samples. Under optimized conditions (Britton–Robinson buffer at pH = 3.29), the obtained linear ranges were 5.00 × 10^−8^–1.00 × 10^−5^ mol/L and 5.00 × 10^−9^–1.00 × 10^−7^ mol/L DIP for DPV and AdSDPV, respectively. The limits of detection of the methods were 1.47 × 10^−8^ mol/L for DPV and 3.96 × 10^−9^ mol/L DIP for AdSDPV.

## 1. Introduction

**Figure 1:**
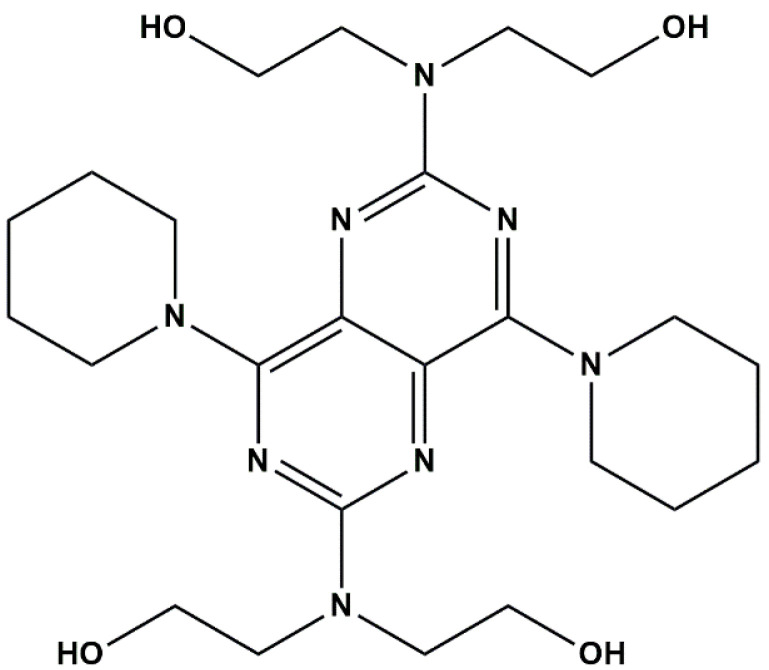
Structural formula of dipyridamole (DIP).

Dipyridamole (Figure 1) (DIP), one of the oldest antithrombotic drugs, used for its capacity to increase coronary blood flow without affecting myocardial oxygen consumption, has undergone many changes due to research results over the years [1]. Initially, it was used with very good outcomes for its coronary vasodilator and anti-anginal properties, and later an increase in its platelet-inhibiting capacity was observed due to concomitant administration with aspirin [2]. It was demonstrated that the co-administration of the two drugs has a better capacity of preventing a recurrent stroke than using them independently. Also, some recent studies suggested that the simultaneous treatment with DIP and aspirin has a better inhibitory effect on colorectal cancer than either monotherapy [2]. Another use of DIP is in conjunction with statins to treat some cancers, such as melanoma, in both veterinary and human medicine [3]. Also, from the point of view of the drugs with which it can be associated, DIP, together with flunarizine and ginkgo leaf extract, can be used with predilection to treat some early forms of vertigo with no additional side effects, improving the patient’s steady state [4]. However, intravenous administration of DIP may induce coronary ischemia, dizziness, abdominal and headache pain as side effects [5].

It was also shown that DIP can be used as a dopant to decrease tiredness and improve efficiency in sports [6]. Nevertheless, DIP bioavailability is limited by its low water-solubility. Hence, a recent study suggested that its apparent solubility and dissolution can be increased by salifying, e.g., forming the corresponding oxalate or malonate [7].

Regarding the study and determination of DIP, several techniques were used to cover both the nature of the sample and the relevant concentration ranges, among them being high performance liquid chromatography [8,9,10], spectrophotometry [11,12] and conductometry [13]. Even so, these methods involved long procedures with complicated sample preparation steps, large amounts of samples/reagents and sometimes, modest sensitivity and selectivity. There are also techniques characterized by lower limits of detection and better selectivity, including fluorimetry [7], phosphorimetry [14] and chemiluminescence [15].

It is well known that the electrochemical methods possess the advantages of reduced analysis time and cost. Hence, they are often the chosen analysis alternatives because they are simple and rapid, being also sensitive and selective, especially when using modified electrodes [16,17,18].

Molecularly imprinted polymers (MIPs) are categorized as a special category of electrode surface modifiers, which contribute to improved sensor performance. Due to their high selectivity and ease of preparation, these materials are commonly employed in this respect using different substrates, among them being most often the glassy carbon electrode (GCE) [19,20,21,22,23,24,25,26] but also some disposable ones such as screen printed electrodes (SPE) [27,28,29,30], pencil graphite electrodes (PGE) [31,32,33,34] or special paper [35], or they may be included in a matrix of electroactive material, as is the case with MIP-based carbon paste electrodes (CPE) [36,37]. An MIP can be synthesized by chemical or electrochemical procedures, the last one being simpler, more rapid and eco-friendly, due to the involvement of a smaller number of chemicals. To create an MIP through a potentiostatic or potentiodynamic electro-polymerization process, it is necessary to use only functional monomers and template molecules. The final procedure for creating 3D cavities in the polymer film that match the shape and dimensions of the analyte is to eliminate the template from the polymeric matrix [38].

Besides excellent selectivity, MIP-based electrodes also allow reaching very low detection limits for very varied analytes, e.g., 3.58 ng/mL β-globulin [19], 4.38 × 10^−15^ mol/L piracetam [33], 1.90 × 10^−13^ mol/L streptomycin [28], 2.09 × 10^−13^ mol/L L-tryptophan [26], 1.67 × 10^−12^ mol/L carbendazim [24], 1.00 × 10^−11^ mol/L cortisol [29], 7.20 × 10^−11^ mol/L chlortetracycline [25], 1.80 × 10^−10^ mol/L bisphenol A [21], etc.

Due to these special performances of the sensors modified with MIP, interest in their development is continuously increasing. To have a clearer and more systematic overview of their synthesis [35,39] and importance, in the literature there are numerous recent reviews structured according to their applicability, e.g., in pharmaceutical and medical analyses [40,41], or to the detected analytes, e.g., antibiotics [38], ascorbic acid, dopamine and uric acid [42], hormones [43,44], biomarkers [35], metal ions [45], etc.

To the best of our knowledge, among the electrochemical sensors reported until now for DIP determination, only three are based on MIPs. In two of them, the polymers were chemically synthesized by complicated procedures [17,18], while the strategy used for the other one [46] was comparable with that applied in this study. The preparation of the MIP-modified PGE (MIP_PGE) tackled in this and our previously published paper [46] was simple, rapid and environmentally friendly due to the fact that it did not involve expensive or toxic materials. Thus, (i) the substrate for MIP deposition was constituted by ordinary graphite pencil lead commonly used for writing; (ii) the monomer was a naturally occurring polyphenol, namely caffeic acid [46] or curcumin (CUR) [47], the yellow phytochemical derived from the plant turmeric, and (iii) the template extraction was performed in ethanol. Even though the two monomers are polyphenols, their chemical structure is different, thus resulting in polymers with different structures. Caffeic acid is a hydroxycinnamic acid while CUR has two phenolic rings, being constituted of two feruloyl moieties bridged by a methylene group. Although, if the two MIP_PGEs have some similarities, the CUR-based MIP_PGE designed for DIP analysis, which features a somewhat broader dynamic range, a lower detection limit and very good selectivity, proved the fact that the possible interferents studied did not present any voltammetric signal in the selected potential window.

The increased consumption of medicines has led to significant enhancements in their production. The pharmaceutical industry has very strict regulations regarding the quality control of both the intermediates and finished products, including the analysis of active principles as well as of the excipients. On the other hand, the drugs are persistent pollutants due to their low solubility, as is the case of DIP [48]. That is why there is a growing need to develop new analysis tools that allow for a fast and reliable screening of the content of pharmaceutical and environmental samples. Therefore, the single-use MIP-modified sensor developed in this study enabled rapid and sensitive DIP determination from both pharmaceuticals and water samples.

## 2. Results

### 2.1. PGE Coating by Electrogenerated Polymeric Film

#### 2.1.1. MIP Preparation—Experimental Conditions

The voltammetric behavior of analytes containing ionizable functional groups depends on the solution pH and, hence, the polymers electrogenerated at the electrode surface. The thickness of the polymeric film, as well as the imprinting degree, influences the performance characteristics of the MIP-modified sensor. On the other hand, these MIP parameters can be adjusted by proper selection of the electro-polymerization conditions, including the supporting electrolyte, the monomer and template concentrations, the number of voltammetric potential cycles and the scan rate.

- The influence of the supporting electrolyte employed in the electro-polymerization process

The effect of the supporting electrolyte employed in the electro-polymerization step on the amplitude of the DIP oxidation signal recorded at the MIP_PGE prepared in the corresponding medium was investigated (Table 1). In this respect, five potential cycles, from 0.000 to 1.000 V, were applied to the PGE. The most intense DIP DPV signal was obtained when the PGE surface was covered with curcumin-based MIP electro-polymerized in 0.2 mol/L NaOH, and therefore, this medium was selected for further investigations. This observation can be correlated with the fact that CUR is soluble in aqueous solutions only at a high pH, and it is most stable in alkaline media (pH > 11.7), where it exists in the fully deprotonated enol form, CUR^3−^ (pK_a3_ = 10.95) [49,50].

**Figure 2:**
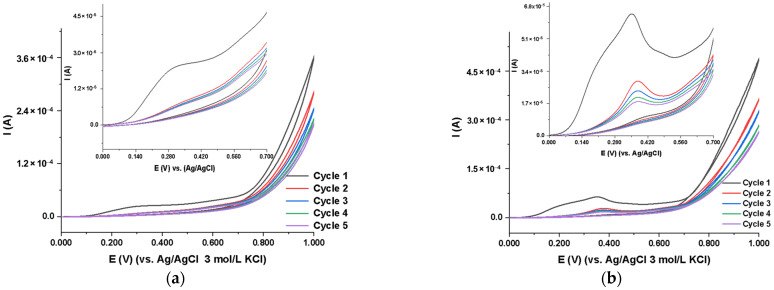
CV curves recorded at PGE for (**a**) 5.00 × 10^−4^ mol/L CUR and (**b**) mixture of 5.00 × 10^−4^ mol/L CUR and 2.50 × 10^−5^ mol/L DIP in 0.2 mol/L NaOH solution, scan rate 0.100 V/s. Inset: expanded section of the potential window containing the CUR and DIP signals.

The PGE surface was modified with polymeric films based either only on CUR (non-imprinted polymer, NIP) or on CUR imprinted with DIP (MIP). The polymers were obtained by electro-polymerization in 0.2 mol/L NaOH. Repetitive cyclic voltammograms recorded for CUR emphasized only an anodic signal at about 0.270 V, whose intensity decreased drastically in the second scan (Figure 2a), becoming even difficult to observe and measure, suggesting the irreversible oxidation of CUR and the covering of the PGE surface with a non-conductive polymer layer (NIP_PGE). In the anodic scan of the first voltammetric cycle recorded in the same conditions for the CUR + DIP mixture (Figure 2b), a more intense oxidation peak (almost double) was observed at more positive potentials (approximately 0.330 V) compared to that obtained for CUR only, which presented also a very badly defined pre-wave at about 0.180 V, indicating that this signal represented the sum of two unresolved peaks (one due to CUR oxidation at less positive potentials and one corresponding to DIP oxidation at about 0.300 V) [46,51]. In the second scan, the anodic peak moved slightly towards higher potential values (0.370 V), and its amplitude decreased by about 60%, while the pre-wave could not be observed anymore. The increase of potential scans resulted in further decrease of the peak amplitude, but the decrease between successive scans became smaller. Considering these observations, one can conclude that in these conditions, the formation of the non-conductive pCUR film at the PGE hindered the electron transfer at the solution–electrode interface and thus, the anodic signal recorded for the CUR + DIP mixture in the subsequent potential scans was due to the irreversible oxidation of DIP, and its intensity decreased due to the fact that the thickness of the non-conductive polymeric film increased with increasing scan numbers.

- The influence of the CUR/DIP ratio employed in the electro-polymerization solution

To get the best conditions for the analyte quantification, it is important to generate an adequate number of cavities in the MIP structure. The effect of CUR content in the electro-polymerization mixture on a DIP voltammetric signal was studied first at the MIP_PGE (Figure S1). To obtain the optimal concentration of monomer, the CUR concentration was varied from 1.25 × 10^−4^ to 6.25 × 10^−4^ mol/L, at a constant template concentration of 1.00 × 10^−5^ mol/L DIP. In these conditions, the highest oxidation signal for DIP was attained at MIP_PGE for 5.00 × 10^−4^ mol/L CUR. This concentration of monomer was kept constant, and the DIP concentration in the electro-polymerization solution was changed between 5.00 × 10^−6^ mol/L and 7.50 × 10^−5^ mol/L (Figure S2). The analyte showed the most intense oxidation peak at the MIP_PGE prepared with an electro-polymerization solution containing 5.00 × 10^−4^ mol/L CUR and 2.50 × 10^−5^ mol/L DIP.

- The influence of the number of cyclic voltammetric scans involved in the electro-polymerization process

The broadness of the polymeric film covering the electrode surface was controlled by varying the number of cyclic scans applied in the electro-polymerization step. To select the optimum number of voltammetric cycles necessary to obtain the highest DPV response for DIP at MIP_PGE, this important parameter of the electro-polymerization step was increased from 3 to 10. The conducted experiments emphasized that the MIP formed at the PGE after 7 potential cycles generated the highest DIP anodic peak (Figure S3).

- The influence of scan rate applied in the e lectro-polymerization process

The proper selection of the scan rate in the electro-polymerization process performed by CV is important because it helps to control the rate with which the polymer layer is grown on the substrate. In our study, the potential applied to the PGE was scanned with different rates varying between 0.050 and 0.300 V/s, maintaining at fixed values the parameters established previously. The highest DIP DPV anodic peak was recorded at MIP_PGE prepared using a scan rate of 0.100 V/s (Figure S4).

#### 2.1.2. Template Removal Conditions

To generate the MIP, the polymerization step must be succeeded by the elimination of the template molecule from the polymer matrix to create the 3D size and shape specific recognition cavities. To remove the DIP from the pCUR matrix, the PGE covered with DIP containing pCUR films was maintained in ethanol for different time periods ranging between 15 to 120 min and the DIP characteristic oxidation peak was monitored by DPV at MIP_PGE in a BRB solution with pH = 3.29. For MIP_PGE immersed longer than 120 min in ethanol no DIP signal was observed indicating that 120 min was the optimal time to completely remove the template from the pCUR + DIP polymer matrix deposited at the PGE.

### 2.2. Characterization of the Electrode Surface

The electroactive surface areas (A_ea_) of PGE, MIP_PGE and NIP_PGE were determined by conducting CV experiments at different scan rates in 1.00 × 10^−4^ mol/L K_4_Fe(CN)_6_ in 0.10 mol/L KCl solution and comparing the slopes of the different regression equations of the I_p_ (A) = f(v^1/2^, (V/s)^1/2^) dependencies with that of the Randles–Sevcik equation [52].

The A_ea_ had the smallest value for PGE (0.0774 cm^2^), followed by NIP_PGE (0.0818 cm^2^) and MIP_PGE (0.0882 cm^2^). In comparison to the bare PGE, both modified electrodes showed an increase in A_ea_, namely of 105.37% and 114.04% for NIP_PGE and MIP_PGE, respectively.

**Figure 3:**
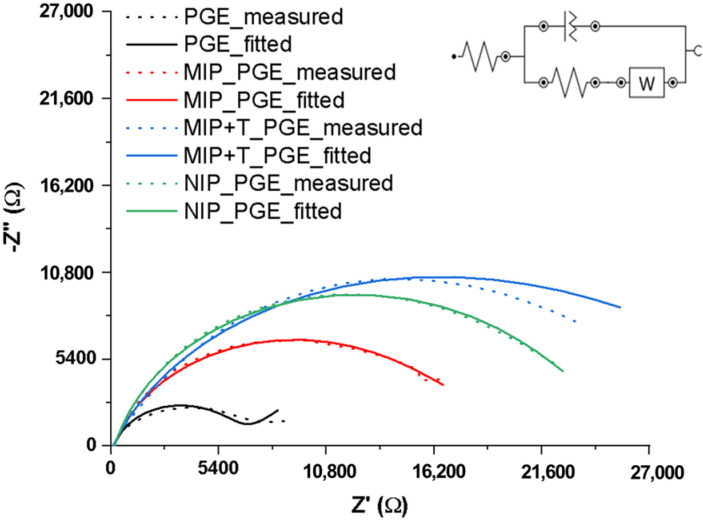
Impedance spectra recorded and fitted for all the tested working electrodes. To obtain the Nyquist plot, 1.00 × 10^−3^ mol/L [Fe(CN)_6_]^4−^/[Fe(CN_6_)]^3−^ in acetate buffer solution with pH = 4.50 was used (DC potential of 0.230 V and frequency in the range 0.1 Hz–10.0 kHz). The equivalent circuit employed for the fitting curve is presented schematically above.

EIS measurements were conducted in an acetate buffer solution with pH = 4.50, using the redox couple [Fe(CN)_6_]^4−^/[Fe(CN_6_)]^3−^ in order to determine which electrode (PGE, MIP + T_PGE, NIP_PGE and MIP_PGE) has the highest charge transfer resistance (R_ct_). The highest value of R_ct_ (29.32 kΩ) was obtained for the MIP + T_PGE (PGE modified with pCUR + DIP still containing template molecules in the polymeric matrix, before DIP extraction). The R_ct_ of the NIP_PGE (electrode covered with pCUR), 24.35 kΩ, was slightly lower than that of MIP + T_PGE but higher than that of the MIP_PGE (MIP-modified electrode) (17.73 kΩ). The lowest value of the charge transfer resistance (6.26 kΩ) was presented by the bare PGE. All the results were extracted from the Nyquist plots (Figure 3).

The results obtained from the impedance spectra were consistent with the behavior of the MIP formation at the PGE by electro-polymerization. The fact that after the first CV cycle the peak intensity decreased certified that the resulting polymer film was non-conductive. The PGE covered with pCUR (NIP_PGE) showed higher resistance than the unmodified electrode but lower than the electrode covered by the pCUR layer containing the analyte. As the DIP was removed from the polymeric matrix, the R_ct_ value decreased due to the formed cavities where the analyte came into contact with the graphite surface, which has a higher conductivity.

### 2.3. DIP Voltammetric Analysis at MIP_PGE

#### 2.3.1. DIP Voltammetric Behavior at MIP_PGE in Supporting Electrolytes with Different pH Values

**Figure 4:**
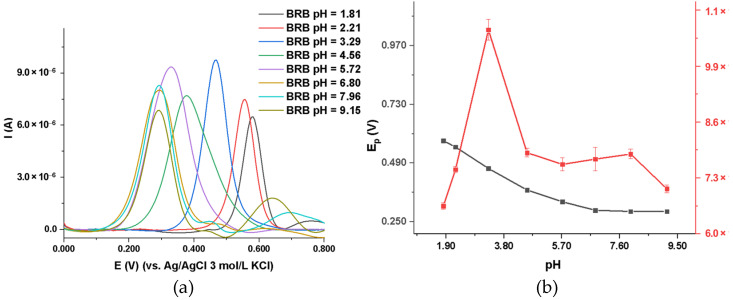
(**a**) DP voltammograms recorded at MIP_PGE for 5.00 × 10^−5^ mol/L DIP in BRB solutions with different pH values and (**b**) the dependencies of DIP oxidation peak potential (E_p_)/current (I_p_) recorded by DPV at MIP_PGE on the pH of the supporting electrolyte.

The pH effect was investigated to characterize the DIP behavior at the MIP_PGE. Therefore, the voltammetric response of the modified disposable electrodes was explored by DPV using as supporting electrolyte BRB solutions (pH between 1.81 to 9.15), either without or with a fixed analyte concentration. As one can observe from Figure 4, the highest DIP anodic peak was recorded at MIP_PGE in a BRB solution with pH = 3.29, so that the next experiments were carried out employing this electrolyte.

When increasing the pH values of the buffer used as supporting electrolyte, DIP anodic peak potential (E_p_) decreased, this change initiated by the proton participation in the oxidation process. The slope of the regression equation E_p_ = −0.0582 × pH + 0.6736 (R^2^ = 0.9758) (Figure 4) applied for the pH range between 1.81 to 6.80, with a value close to the theoretical one from the Nernst relation, suggested that DIP electro-oxidation implied an equal number of protons and electrons, this result being consistent with data previously reported in the literature [6]. The E_p_ = f (pH) dependence presented a break at pH values between 6 and 7, thus allowing the estimation of the DIP pKa value, which was found to be 6.48. This result is consistent with the data given in the literature [53].

Taking into account CUR dissociation constants (pK_a1_ = 7.43, pK_a2_ = 9.55 and pK_a3_ = 10.95) and the distribution of its species in solution, as reported in the literature [50], one can consider that in supporting electrolytes with pH, less than 7.40 pCUR was neutral, while at higher pH values, it becomes ionized, being negatively charged.

#### 2.3.2. DIP Voltammetric Behavior at Different Potential Scan Rates at MIP_PGE

The effect of scan rate on DIP voltammetric response at MIP_PGE was studied to bring insight into the nature of DIP electrode processes using this new type of working electrode and to find out if DIP could be adsorbed at the electrode surface in order to exploit this behavior in the quantitative determination of the analyte at lower concentration levels.

**Figure 5:**
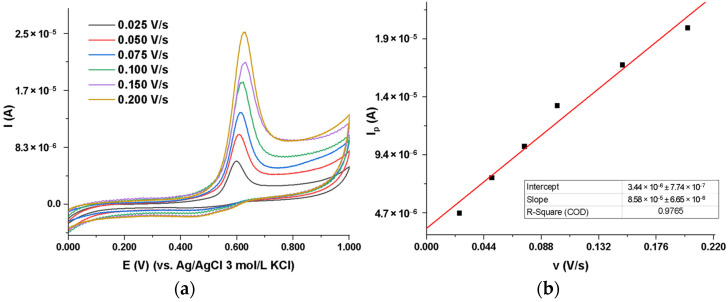

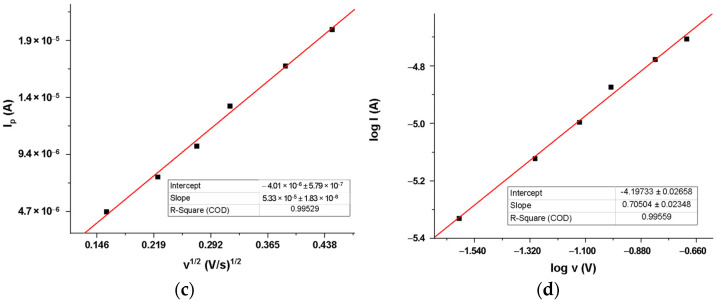
(**a**) CV curves recorded at different scan rates at MIP_PGE for 1.50 × 10^−4^ mol/L DIP in BRB solution with pH = 3.29; and the dependencies (**b**) I_p_ = f(v), (**c**) I_p_ = f (v^1/2^) and (**d**) log I_p_ = f (log v).

In this respect, CV curves were recorded at scan rates varied from 0.025 to 0.200 V/s (Figure 5a), using as supporting electrolyte a BRB solution with pH = 3.29, where DIP presented the highest oxidation signal.

The usual dependencies of the peak current on the scan rate were evaluated (Figure 5b–d). It is known that a linear variation of the peak current with the scan rate indicates an adsorption-controlled electrode process, while a diffusion-limited one is characterized by a linear increase of the peak current with the square root of the scan rate. Therefore, starting from the fact that the correlation coefficient of the I_p_ = f(v^1/2^) dependence was only slightly better than that of the I_p_ = f(v) one, and the slope of the regression equation describing the log I_p_ = f(log v) variation was 0.705 (Table 2), a value situated between that corresponding to a diffusion controlled process (0.500) and 1.000, characteristics for a surface-confined electrode process, it was concluded that DIP electro-oxidation at MIP_PGE was determined by both diffusion and adsorption phenomena.

From Figure 5, one can also observe that increasing the scan rate resulted in a shift of DIP anodic peak towards more positive potentials. This behavior is characteristic of irreversible electrode processes. Comparing the slope of the E_p_ = f(log v) dependence, described by the equation E_p_ (V) = 0.0325 × log v + 0.6503 (R^2^ = 0.9826) with the term $\left(\frac{2.303RT}{\alpha nF}\right)logv$ from the Laviron relation [54], where v = scan rate (V × s^−1^), α = charge transfer coefficient, which is 0.5 for an irreversible process, T = absolute temperature (298 K), R = universal gas constant (8.314 J × K^−1^ × mol^−1^) and F = Faraday constant (96,480 C × mol^−1^, the number of transferred electrons was calculated to be n = 3.64, which could be approximated to 4. Correlating this result with the conclusion of the pH study, one can state that DIP electrooxidations at MIP_PGE involved 4 electrons and 4 protons.

**Scheme 1:**
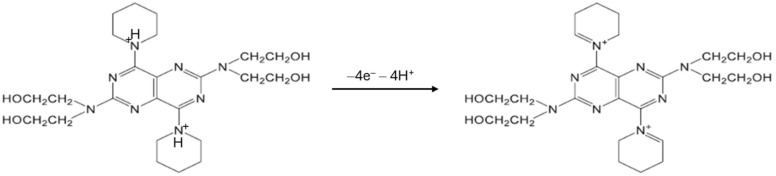
Possible mechanism for DIP electrooxidation at MIP_PGE in BRB solution with pH = 3.29.

DIP is a weak base with pKa = 6.48, so that in the acidic media in which the cyclic voltammograms were recorded, it existed in protonated form. Having this in mind, and combining our results with the tentative mechanism previously published for DIP electrooxidation at bare PGE in PBS at pH = 7.00 [23], where the molecule is predominantly in neutral form, one can consider that DIP electrooxidation at MIP_PGE in a BRB solution with pH = 3.29 could be ascribed to the reaction suggested in Scheme 1:

#### 2.3.3. MIP_PGE Voltammetric Response to DIP Concentration

**Figure 6:**
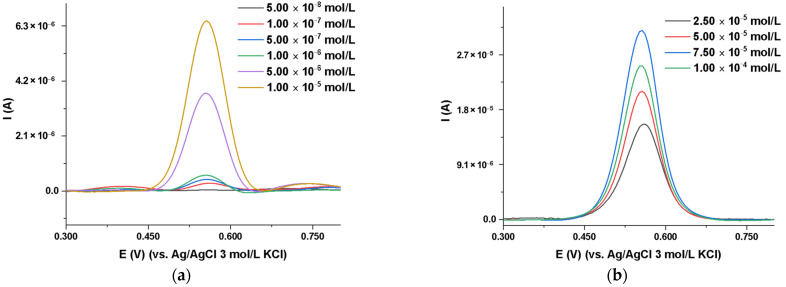
DP voltammograms recorded at MIP_PGE for BRB pH 3.29 solutions containing different DIP concentrations between (**a**) 5.00 × 10^−8^–5.00 × 10^−6^ and (**b**) 1.00 × 10^−5^–1.00 × 10^−4^ mol/L.

The way in which the MIP_PGE responded to the variation of DIP concentrations was investigated by DPV in the BRB solution with pH = 3.29 with different analyte concentrations from 5.00 × 10^−8^ to 1.00 × 10^−4^ mol/L (Figure 6). The oxidation peak current grew with DIP concentration increments showing two dynamic linear domains (Table 3).

In some types of matrices, such as biological fluids or environmental samples, DIP may be present at very low concentrations. Therefore, considering that the DIP electrode process also has an adsorptive component, the possibility to accumulate the analyte at the MIP_PGE surface was investigated to allow DIP quantification at lower concentration levels compared to DPV. In this respect, the accumulation potential (E_acc_) and the accumulation time (t_acc_) were optimized. In the first step, the E_acc_ was changed from −0.500 V to 0.200 V by keeping the t_acc_ constant at 30 s. The most intense DIP anodic signal was observed for the applied E_acc_ of −0.400 V (Figure S5a). Afterward, the optimal t_acc_ was determined by varying this parameter between 15 and 90 s while keeping the E_acc_ constant at −0.400 V (Figure S5b).

Due to the small surface of the electrode, an accumulation time of 30 s was enough to get the highest DIP oxidation peak at MIP_PGE. For accumulation times exceeding 30 s, a decrease in the analyte oxidation peak was recorded, most probably due to the saturation of the MIP_PGE surface with analyte molecules.

**Figure 7:**
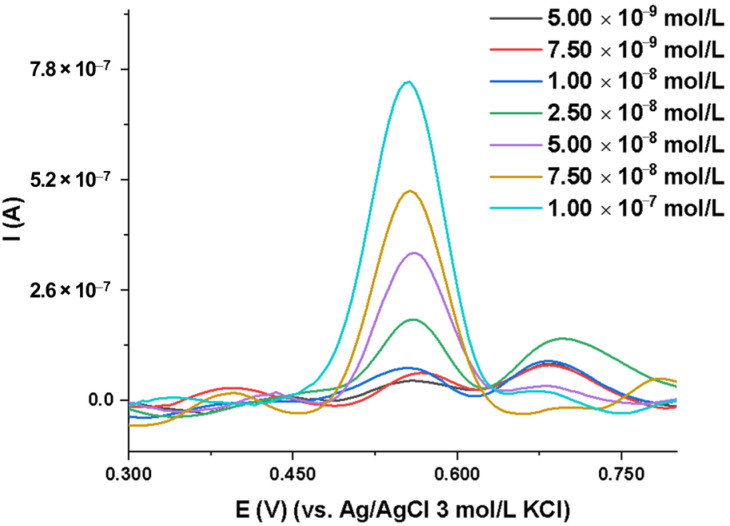
AdSDP voltammograms recorded at MIP_PGE for different DIP concentrations in BRB solution with pH = 3.29; t_acc_ 30 s; E_acc_ −0.400 V.

Using the optimized accumulation conditions E_acc_ −0.400 V and t_acc_ 30 s, the influence of the DIP concentration on the electrode response was investigated through adsorptive stripping differential pulse voltammetry (AdSDPV). In this case, the linear variation of the peak current intensity with the analyte concentration from 5.00 × 10^−9^ to 1.00 × 10^−7^ mol/L, DIP (Figure 7) was described by the regression equation I_p_ (A) = 7.2127 × C_DIP_ (mol/L) + 1.000 × 10^−8^ (R^2^ = 0.9994).

#### 2.3.4. Performance Characteristics of the Developed Voltammetric Methods at MIP_PGE

The limits of detection (LOD) and quantification (LOQ) of the DPV and AdSDPV methods developed for DIP quantification at pCUR-based MIP_PGE were assessed (Table 4) using the relations LOD = 3.3 × σ_c,min_/S and LOQ = 10.0 × σ_c,min_/S, where σ_c,min_ represents the standard deviation of the signal obtained for the lowest concentration of the linear range and S the slope of the calibration line.

The pCUR-based MIP_PGE developed in this study showed comparable performances with the sensors reported in the literature, such as the syringe carbon paste electrode (SCPE) [13], the maghemite nanoparticles carbon paste electrode [55] and the boron-doped diamond electrode (BDDE) [56], while the NiCo_2_O_4_/NiO@MOF-5/rGO/GCE (MOF—metal organic framework, rGO—reduced graphene oxide) had a wider linear domain (2.00 × 10^−8^–5.50 × 10^−4^ mol/L) but almost similar LOD (2.80 × 10^−9^ mol/L) [57] (Table 5). MIP-modified electrodes were a CPE and a PGE that showed linear ranges of 2 and 1.5 orders of magnitude and LODs of 9.90 × 10^−10^ and 8.67 × 10^−9^ mol/L DIP, respectively [17,46]. The lowest LODs were published for the MIP-modified Fe_3_O_4_@Au/amine-multi-walled carbon nanotubes magnetic GCE (5.95 × 10^−11^ mol/L DIP) [17], followed closely by a Nafion modified GCE (8.00 × 10^−11^ mol/L), but the last one needed 4 min for the analyte accumulation at the electrode surface [58]. However, despite the fact that other electrochemical sensors presented lower LODs, the pCUR-based MIP_PGE had the main advantages of being disposable, easy to prepare and involving a very low accumulation time (30 s).

#### 2.3.5. Reproducibility

The electrode-to-electrode reproducibility of the developed MIP_PGE was assessed at the lowest, intermediate and highest DIP concentration, according to a linear range (Table 6) using both DPV and AdSDPV. For each concentration, 10 measurements were performed, each using a newly manufactured MIP_PGE, as the sensor is disposable. The RSD% values obtained for each concentration were within the accepted range and showed that the developed and optimized sensor is reproducible and stable for the analysis of DIP [61].

#### 2.3.6. Stability

**Figure 8:**
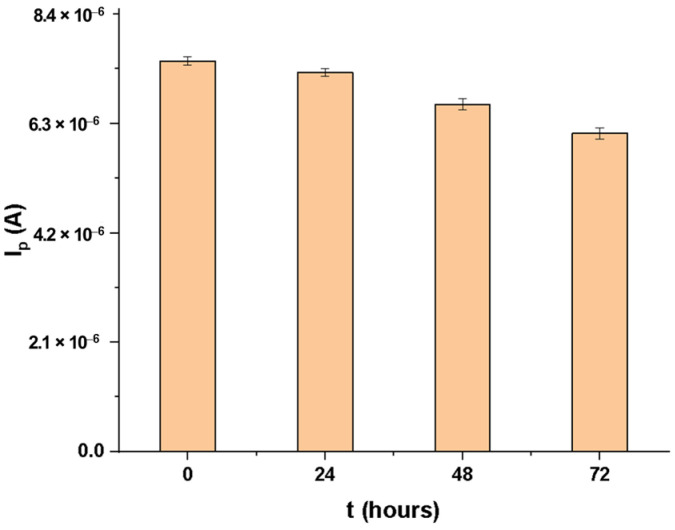
The variation of the anodic peak current recorded at MIP_PGE for 1.00 × 10^−5^ mol/L DIP in BRB solution with pH = 3.29 at different periods of time, including 0, 24, 48 and 72 h after the sensor preparation.

The MIP_PGEs were kept at room temperature in separated vials, and the stability of their voltammetric response was studied for 72 h by measuring the height of DIP anodic peak current. The results schematically shown in Figure 8 represent the mean value obtained from five different electrodes, each being tested at the specified times. A decrease of 7.29% and 18.53% of the initial peak height was observed after 24 h and 72 h, respectively (Figure 8).

#### 2.3.7. Determination of the Imprinting Factor

To evaluate the ability of the CUR-based MIP_PGE to recognize the chemical structure of DIP, the imprinting factor (IF) was assessed using the following equation:

IF = S_MIP_PGE_/S_NIP_PGE_,

where S_MIP_PGE_ and S_NIP_PGE_ represent the sensitivity of the MIP_PGE and of the NIP_PGE, respectively.

The value obtained for the IF was 2.15, indicating an efficient molecular recognition. Moreover, the NIP_PGE linearity range (3.00 × 10^−7^–1.00 × 10^−4^ mol/L) comprised higher concentrations than the one obtained for MIP_PGE, justifying once more the use of the MIP_PGE.

#### 2.3.8. Interferences

Chemical species commonly found in biological fluids such as glucose, ascorbic acid, aspirin, acetaminophen and phenylalanine, each at a concentration of 1.00 × 10^−5^ mol/L, were tested as interferents in DIP (2.00 × 10^−6^ mol/L) voltammetric analysis at the MIP-modified electrode, considering a tolerance limit up to ±10% the signal change. The possible interferent species were selected considering the following three perspectives: (i) aspirin and acetaminophen can be found together in pharmaceutical preparations [62], (ii) glucose is administered intravenously alongside DIP [63], and (iii) phenylalanine is contraindicated as a simultaneous medication with DIP [64].

**Figure 9:**
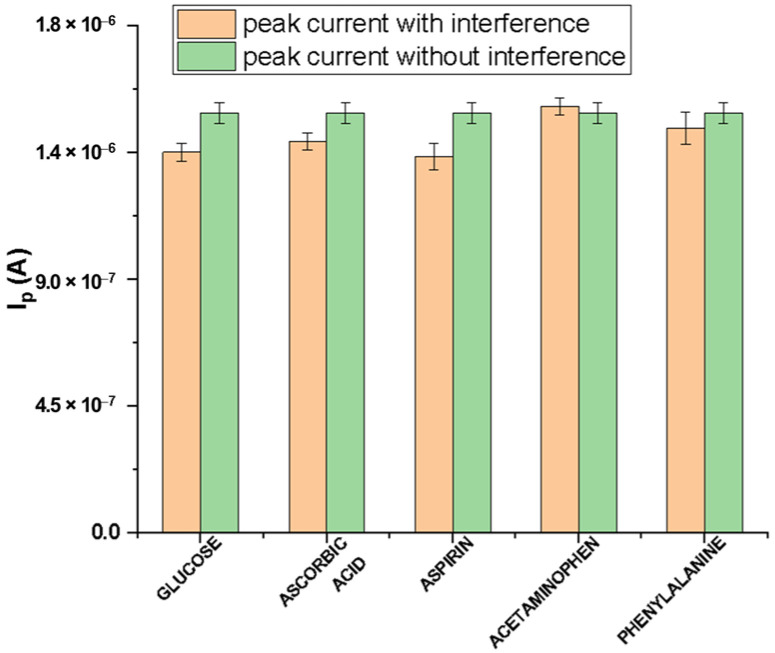
DPV peak currents recorded at MIP_PGE for 2.00 × 10^−6^ mol/L DIP in BRB solution with pH = 3.29, without and with a 50-fold excess of different possible interfering species.

The experimental results showed that glucose, ascorbic acid, aspirin and phenylalanine decreased DIP DPV peak current with 9.04%, 9.33%, 8.99% and 9.66%, while acetaminophen slightly increased it with 1.01% (Figure 9).

#### 2.3.9. DIP Quantification in Tablets/Tap Water Samples Using the MIP-PGE

The analytical applicability of the MIP_PGE was tested by quantifying DIP from pharmaceutical tablets and tap water. The samples were properly processed and analyzed in a BRB solution with pH = 3.29, applying the successive standard addition method to reduce the eventual influence of the matrix components.

**Figure 10:**
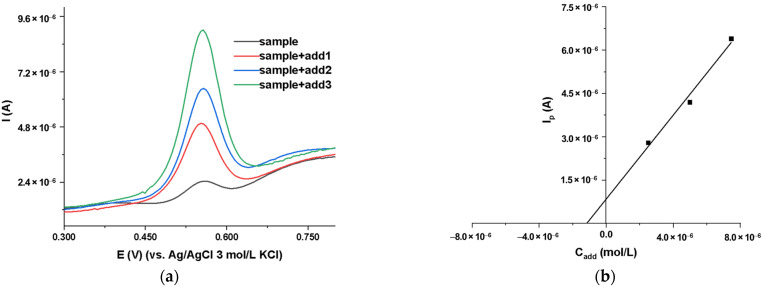
(**a**) DP voltammograms for 10 mL DIPIRIDAMOL tablets working solution in BRB pH = 3.29, recorded at MIP_PGE. The initial sample and the 3 × 0.025 mL addition of 1.00 × 10^−3^ mol/L DIP is also presented; (**b**) the dependence of the DIP oxidation signal on the C_add_ DIP.

The DP voltammograms of the DIPIRIDAMOLE tablets’ working solution, recorded each time on a new MIP_PGE, consistently displayed only the characteristic DIP oxidation signal (~0.500 V). This suggested that in these conditions, the tablets’ excipients were electroinactive. DIP anodic peak currents, measured before and after each addition of analyte standard solution (Figure 10a), increased linearly with the rising concentrations of added DIP (Figure 10b). These measurements were used to determine the DIP content in the pharmaceutical sample, considering the performed dilutions. The results, presented in Table 7, confirm the effectiveness of the developed DPV at MIP_PGE method for quantifying DIP in pharmaceutical samples. Based on DIP intrinsic fluorescence properties, to validate the DPV at MIP_PGE method, the same samples were also analyzed by spectrofluorometry.

Tap water samples diluted at 1:1 with a BRB solution at pH = 3.29 were enriched with DIP standard solution to a total concentration of 2.50 × 10^−7^ mol/L, 5.00 × 10^−7^ mol/L and 7.50 × 10^−7^ mol/L and analyzed by DPV at MIP_PGE. The anodic peak current recorded for the spiked water samples had encountered a linear increase. Using the calibration curve, the recovery was estimated for each concentration level (Table 8). The obtained mean recovery (R ± SD (%)) was 105.78 ± 0.0032. The same samples were analyzed by spectrofluorometry, employed as a standard method. In this case, the mean percentage recovery of added DIP was (102.47 ± 2.97) %.

## 3. Experimental

### 3.1. Reagents

Dipyridamole (≥98.0%, HPLC), curcumin (94.0%), ethanol (≥95.0% ACS spectrophotometric grade), Na_2_HPO_4_ × 2H_2_O and KH_2_PO_4_ (p.a., ACS reagent), acetic acid (≥99.7%, glacial, ACS reagent), sodium acetate (≥99.0%, ACS reagent), L-phenylalanine (≥98%, reagent grade), acetylsalicylic acid (≥99.0%), NaOH (pellets), urea (99.0–100.5%, ACS reagent), glucose (≥99.5%, GC), citric acid (99.0%), ascorbic acid (reagent grade), acetaminophen (98.0–102.0%), H_3_BO_3_ (1 g per tablet) and H_3_PO_4_ (85 wt% in H_2_O) were purchased from Merck, and DIPIRIDAMOL tablets (25 mg of DIP/tablet), manufactured by S.C. Zentiva S.A., Bucharest Romania, were bought from a drug store.

### 3.2. Solutions

Stock solutions of the compounds involved in the polymerization process were 1.00 × 10^−3^ mol/L DIP and 5.00 × 10^−3^ mol/L CUR. From these, successive dilutions were made to obtain working solutions in the corresponding supporting electrolyte. For pH influence investigation, Britton–Robinson buffer (BRB) solutions with variable pH values from 1.81 to 9.15 were used.

### 3.3. Instrumentation

A PGSTAT 12 potentiostat/galvanostat (Metrohm Autolab, The Netherlands) connected to both a voltammetric cell and a PC running the General Purpose Electrochemical System (GPES) 4.9. software was employed for the electrochemical measurements. The voltammetric cell consisted of the following three electrodes: the working electrode (PGE bare or modified with MIP), Ag/AgCl/KCl (3.00 mol/L) as reference and a Pt wire as counter electrode (both from Metrohm, The Netherlands), respectively. The working electrode consisted of a Rotring graphite mine with HB hardness and a 0.50 mm diameter, bought from a local bookstore. The procedure of making a proper electrode was previously described [65]. By always immersing 1.00 cm of the graphite lead into the voltammetric solution, a reproducible PGE geometrical electroactive surface area of 15.86 mm^2^ was ensured.

A PGSTAT302N potentiostat/galvanostat (Metrohm Autolab, Utrecht, The Netherlands) working under Nova 1.11, connected to a three-electrode cell was used for the electrochemical impedance (EIS) measurements.

The solutions pH values were measured with a conventional combined glass pH-selective electrode connected to a pH/mV/°C-meter (Consort P901 Scientific Instrument, Namur, Belgium).

Fluorescence measurements were performed in a fluorescence cell with a 10.00 mm path employing a research-grade spectrofluorometer FP-6500 (Jasco, Tokyo, Japan) connected to a desktop running the software Spectra Manager.

### 3.4. Procedures

**Scheme 2:**
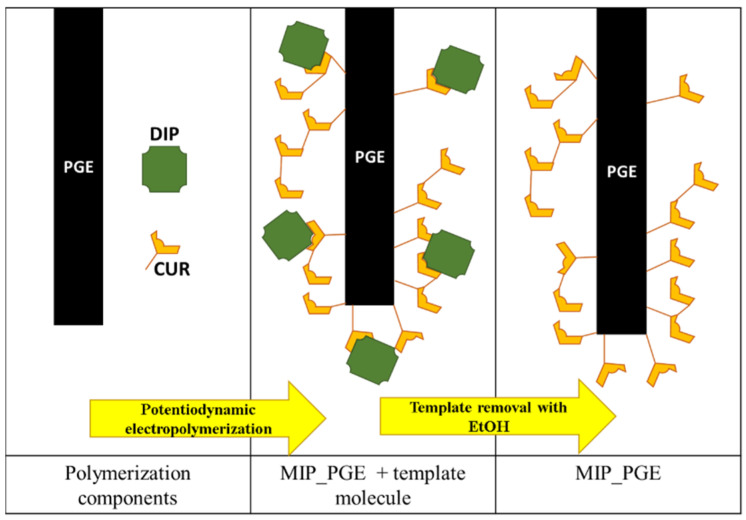
Schematic representation of the steps involved in the preparation of the MIP_PGE.

The PGE surface was modified with a polymeric film electrogenerated by potentiodynamic polymerization of a mixture consisting of the monomer CUR and the template DIP in 0.2 mol/L NaOH. If not stated otherwise, the polymerization was performed by cycling 7 times the potential applied to the PGE, from 0.000 to 1.000 V, with a scan rate of 0.100 V/s and step size potential of 0.0244V. MIP_PGE was obtained by removing the DIP molecules from the poly (curcumin) (pCUR) matrix deposited at the PGE surface. This was performed by immersing the polymer-coated PGE in ethanol for 2 h until the DIP oxidation signal was no longer observed in the voltammograms recorded in the supporting electrolyte (BRB, pH = 3.29) (Scheme 2).

CUR polymerization in the same above mentioned conditions, but without DIP, resulted in the fabrication of a non-imprinted poly (curcumin)-modified PGE (NIP_PGE) employed for comparison purposes.

Differential pulse voltammetry (DPV) curves were obtained in the potential range of 0.000–1.200 V, applying a modulation amplitude of 75 mV, a step potential of 4.94 mV, an interval time of 0.1 s and a modulation time of 0.002 s.

The impedance measurements were intended to determine the charge transfer resistance (R_ct_) of the electrochemical sensors used as working electrodes in this work. The conditions used were a frequency in the range 0.1 Hz–10.0 kHz and a DC potential of 0.230 V. All the experiments were conducted in an acetate buffer solution with pH = 4.50, containing 1.00 × 10^−3^ mol/L [Fe(CN)_6]_]^4−^/[Fe(CN_6_)]^3−^. The results were represented as Nyquist plots and interpolated using Randles equivalent circuit in order to determine R_ct_. The other important parameters used in this respect were Rs, (the electrolyte resistance), Q (the constant phase element related to the double layer capacitance) and W (the Warburg impedance employed to simulate the mass-transport effects in the solution).

From the fine powder obtained after grounding 10 tablets of DIPIRIDAMOL 25 mg, an accurate weight amount equivalent for the preparation of a 50 mL solution of 1.00 × 10^−3^ mol/L DIP was dissolved in approximately 25 mL ethanol. To assure the complete dissolution of the active principle, the resulting suspension was sonicated for 30 min and passed through a filter (pore size 22 μm) directly into a 50 mL volumetric flask, which was brought to the mark with ethanol, resulting in the DIPIRIDAMOL tablets stock solution. From this, the DIPIRIDAMOL tablets working solution with a theoretical concentration of 1.00 × 10^−6^ mol/L DIP was prepared just before the analysis by proper dilution with BRB, pH = 3.29. To minimize the matrix interferences, the standard addition method was applied, consisting of 3 successive additions of 0.025 mL of 1.00 × 10^−3^ mol/L DIP stock solution. Thus, DIP DPV oxidation peak currents (I_p_) recorded at the MIP_PGE for the DIPIRIDAMOL tablets working solution (10 mL) before and after each addition of DIP stock solution were employed to construct the calibration graph, representing the peak currents I_p_ (A) vs. the DIP concentration added into the analyzed solution (C_add_, mol/L). The DIP concentration in the DIPIRIDAMOL tablets working solution was assessed from the regression equation of the previously obtained I_p_ (A) = (C_add_, mol/L) dependence. The content of the DIPIRIDAMOL tablets was estimated from the resulting DPV data, considering the mass of tablets powder taken for the analysis and all dilutions necessary to obtain the voltammetrically investigated solution.

Spectrofluorometric analysis of DIP was performed in a BRB solution with pH = 7.00 at an excitation wavelength of 286 nm and an emission wavelength of 488 nm.

## 4. Conclusions

This study presented the development of an MIP-based electrochemical sensor applied to DIP voltammetric analysis. Using CUR, the polyphenolic compound responsible for the yellow color of turmeric, as a monomer and DIP as a template molecule and a potentiodynamic electro-polymerization procedure, a cost-effective, selective sensor for DIP detection was easily obtained, employing small amounts of less- or even non-toxic reagents. The template molecule removal from the MIP_PGE was performed by immersing the electrode in ethanol for 2 h. After optimization of the supporting electrolyte, DPV and AdSDPV techniques were used for DIP analysis in BRB solutions at pH = 3.29, attaining LODs of 1.47 × 10^−8^ mol/L and 3.96 × 10^−9^ mol/L, respectively. Both techniques presented wide linear ranges of 5.00 × 10^−8^–1.00 × 10^−5^ mol/L and 5.00 × 10^−9^–1.00 × 10^−7^ mol/L, respectively. The reproducibility of the new developed disposable sensor was estimated at three DIP concentration levels applying both DPV and AdSDPV. The obtained RSD values were within the accepted limits, suggesting a good reproducibility of the MIP_PGE. The electrode was stable up to 24 h, but this is not a major inconvenience because the electrode is disposable and can be easy prepared just before use. Interference studies emphasized that common biologically important compounds did not affect the DIP anodic response. The MIP_PGE allowed the DIP quantification from real samples (tablets and spiked tap water) with good recoveries approaching 100%. Comparing the performance characteristics of the sensor developed here with those of the electrodes previously reported for DIP analysis, one can conclude that in terms of linear range and LOD, the CUR-based MIP_PGE has similar performances with many of them, but it has the main advantages of facile, eco-friendly and cost-effective preparation, as well as short analysis times. Summing up all of this, the MIP_PGE can be used for rapid and efficient DIP quantification and could be a simple and effective tool for the quality control analysis of pharmaceuticals and water samples. However, future investigations will be directed to shorten the time necessary for the total template removal from the polymer matrix and to enhance the sensor stability period, especially for non-disposable electrodes.

## Figures and Tables

**Table 1:** Anodic peak currents, I_p_ (A), recorded for 1.00 × 10^−5^ mol/L DIP in a BRB pH = 3.29 solution by DPV at PGE modified with MIP prepared in different electro-polymerization media containing 5.00 × 10^−4^ mol/L CUR and 2.50 × 10^−5^ mol/L DIP.

Supporting Electrolyte Employed in the Electro-Polymerization Process	I_p_ (A)
0.1 mol/L HCl Phosphate buffer solution (PBS) pH = 7.00 0.2 mol/L NaOH	5.76 × 10^−6^
	6.98 × 10^−6^
	7.51 × 10^−6^

**Table 2:** The regression equations of the dependencies of the DIP anodic peak currents (I_p_, A) on the potential scan rate (v, V/s) corresponding to the cyclic voltammograms presented in Figure 6.

Dependence	Regression Equation
I_p_ = f(v)	I_p_ = 8.581 × 10^−5^ × v + 3.4420 (R^2^ = 0.9765)
I_p_ = f (v^1/2^)	I_p_ = 5.327 × 10^−5^ × v^1/2^ − 4.0140 × 10^−6^ (R^2^ = 0.9953)
log I_p_ = f (log v)	log I_p_ = 0.705 × log v − 4.1973 (R^2^ = 0.9956)

**Table 3:** Regression equations of the I_p_ (A) = f (C_DIP_) variations obtained by DPV at MIP_PGE.

Concentration Range (mol/L)	Regression Equation	Correlation Coefficient
5.00 × 10^−8^–1.00 × 10^−5^	I_p_ (A) = 0.7341 × C_DIP_ (mol/L) + 2.000 × 10^−8^	(R^2^ = 0.9994)
2.50 × 10^−5^–1.00 × 10^−4^	I_p_ (A) = 0.2016 × C_DIP_ (mol/L) + 1.000 × 10^−5^	(R^2^ = 0.9997)

**Table 4:** The detection (LOD) and quantification (LOQ) limits of the voltammetric methods at MIP_PGE developed for DIP quantification.

Technique	LOD (mol/L)	LOQ (mol/L)
DPV	1.47 × 10^−8^	6.80 × 10^−8^
AdSDPV	3.96 × 10^−9^	2.89 × 10^−8^

**Table 5:** Comparison of the performances of electrochemical sensors and methods reported for DIP quantification.

Electrode	Technique/Conditions	Linear Range (mol/L)	LOD (mol/L)	Sample	Mean Recovery (%)	Ref.
HMDE	SWV/PBS pH = 3.00	1.28 × 10^−6^–7.02 × 10^−6^	1.88 × 10^−8^	Tablets	100.8	[11]
				Injections	103.4	
HMDE	CAdSSWV/BRB pH = 8.00; E_acc_ = −1.000 V; t_acc_ = 300 s	9.00 × 10^−9^–5.00 × 10^−6^	4.00 × 10^−11^	Human serum	98.38	[59]
SME	AdSV/0.05 mol/L NaOH pH = 12.70 + 10% (*v*/*v*) ethanol; E_acc_ = 1.200 V; t_acc_ = 60 s	5.00 × 10^−9^–1.00 × 10^−8^	t_acc_ = 300 s; 1.00 × 10^−9^	Tablets	105.00	[60]
				Injections	92.00	
BDDE	DPV/BRB pH = 3.00 SWV/BRB pH = 3.00	1.00 × 10^−7^–5.00 × 10^−6^	4.00 × 10^−8^	Capsules	DPV: 95.80 SWV: 96.70	[56]
		1.00 × 10^−7^–6.00 × 10^−6^	6.00 × 10^−8^	Human urine	DPV: 103.85 SWV: 97.35	
CPE	DPV/6.00 × 10^−5^ mol/L CTAB; t_acc_ = 120 s	5.95 × 10^−8^–2.38 × 10^−7^	1.98 × 10^−8^	Tablets	102.23	[6]
SCPE	SWV/PBS pH = 5.20; E_acc_ = 0.580 V; t_acc_ = 100 s	8.00 × 10^−8^–3.00 × 10^−5^	2.00 × 10^−8^	Tablets	98.80	[13]
PGE	DPV/PBS pH = 7.00	5.00 × 10^−7^–2.50 × 10^−4^	1.21 × 10^−7^	Tablets	200.67	[51]
Nafion-GCE	AdSV/BRB pH = 1.70; E_acc_ = 0.000 V; t_acc_ = 60 s	1.00 × 10^−9^–8.00 × 10^−8^	t_acc_ = 240 s 8.00 × 10^−11^	Human serum	100.70	[58]
NiCo_2_O_4_/NiO@MOF-5/rGO/GCE	DPV	2.00 × 10^−8^–5.50 × 10^−4^	2.80 × 10^−9^	Free-drug plasma	100.10	[57]
				Urine	98.10	
AgNP/MWCNT/PANI/HFME	DPV/PBS pH = 6.50	1.00 × 10^−8^–5.00 × 10^−5^	1.00 × 10^−9^	Tablets	100.30	[48]
				Human serum	103.00	
MIP_Fe_3_O_4_@Au-H_2_N-MWCNT/MGCE	DPV/PBS pH = 3.00	9.91 × 10^−10^–3.76 × 10^−6^	5.95 × 10^−11^	Human serum	99.23	[17]
γ-Fe_2_O_3__CPE	DPV/BRB pH = 2.00; t_acc_ = 75 s	1.00 × 10^−8^–5.70 × 10^−6^	5.00 × 10^−9^	Tablets	98.10	[55]
		5.70 × 10^−6^–5.00 × 10^−5^		Human serum	99.30	
MIP_CPE	AdSDPV/PBS pH = 3.00; E_acc_ = −0.200 V; t_acc_ = 120 s;	1.98 × 10^−9^–2.18 ×10^−7^	9.90 × 10^−10^	Tablets	98.60	[18]
				Human serum	97.77	
MIP_PGE	DPV/PBS pH = 7.00 AdSDPV/PBS pH = 7.00; E_acc_ = 0.200 V; t_acc_ = 30 s	1.00 × 10^−7^–1.00 × 10^−5^	2.04 × 10^−8^	Tablets	105.16	[46]
		1.00 × 10^−8^–5.00 × 10^−7^	8.67 × 10^−9^			
MIP_PGE	DPV/BRB pH = 3.29 AdSDPV BRB pH = 3.29; E_acc_ = −0.400 V; t_acc_ = 30 s	5.00 × 10^−8^–1.00 × 10^−5^	1.47 × 10^−8^	Tablets Tap water	104.67 105.78	This work
		5.00 × 10^−9^–1.00 × 10^−7^	3.96 × 10^−9^			

AdSV: adsorptive stripping voltammetry; AgNP/MWCNT/PANI/HFME: silver/multiwall carbon nanotube/polyaniline nanocomposite hollow fiber membrane electrode; CAdSSWV: cathodic adsorptive stripping square wave voltammetry; CTAB: cetyltrimethyl ammonium bromide; Fe_3_O_4_@Au-H_2_N-MWCNT/MGE: Fe_3_O_4_@Au nanoparticles immobilized on amine-multi-walled carbon nanotubes magnetic glassy carbon electrode; HMDE: hanging mercury drop electrode; SME: static mercury electrode; SWV: square wave voltammetry.

**Table 6:** Reproducibility results (expressed as RSD%), of the voltammetric methods at pCUR-based MIP-modified PGE for DIP analysis using a BRB solution with pH = 3.29 as supporting electrolyte.

DPV		AdSDPV	
DIP Concentration (mol/L)	RSD% *	DIP Concentration (mol/L)	RSD% *
1.00 × 10^−7^	7.90	1.00 × 10^−8^	8.88
1.00 × 10^−6^	3.72	7.50 × 10^−8^	5.91
1.00 × 10^−5^	3.09	5.00 × 10^−7^	4.06

* Relative standard deviation.

**Table 7:** The results obtained for the estimation of the DIP content of pharmaceutical tablets by DPV at MIP_PGE and spectrofluorometry.

Technique	Tablet’s DIP Content (mg)		
	Claimed by Manufacturer	Found ± SD *	Mean Recovery ± SD * (%)
DPV at MIP_PGE	25.00	26.16 ± 0.47	104.66 ± 0.025
Spectrofluorometry	25.00	26.17 ± 0.77	104.67 ± 3.06

* SD: standard deviation (n = 3).

**Table 8:** Results obtained by DPV at MIP_PGE and spectrofluorometry for DIP recovery for each concentration level added to tap water samples (n = 3).

DIP Concentration (mol/L)				
Added	Found by		Mean Recovery %	
	DPV	Spectrofluorometry	DPV	Spectrofluorometry
2.50 × 10^−7^	2.64 × 10^−7^	2.58 × 10^−7^	105.55	103.20
5.00 × 10^−7^	5.31 × 10^−7^	5.25 × 10^−7^	106.14	105.00
7.50 × 10^−7^	7.92 × 10^−7^	7.44 × 10^−7^	105.65	99.20

## Data Availability

Data is contained within the article or Supplementary Materials.

## Supplementary Materials

The following supporting information can be downloaded at https://www.mdpi.com/article/10.3390/molecules29194630/s1. Figure S1: The effect of CUR concentration in the polymerization mixture on the DIP oxidation signal recorded by DPV in a BRB solution with pH = 3.29 at MIP_PGE. Polymerization conditions: C_DIP_ = 1.00 × 10^−5^ mol/L; supporting electrolyte 0.2 mol/L NaOH; 5 voltammetric cycles between 0.000 and 1.000 V; scan rate 0.100 V/s. Figure S2: The effect of DIP concentration in the polymerization mixture on the DIP oxidation signal recorded by DPV in a BRB solution with pH = 3.29 at MIP_PGE. Polymerization conditions: C_CUR_ = 5.00 × 10^−5^ mol/L; supporting electrolyte 0.2 mol/L NaOH; 5 voltammetric cycles between 0.000 and 1.000 V; scan rate 0.100 V/s. Figure S3: Comparison of the DIP oxidation signal recorded by DPV in a BRB solution with pH = 3.29 at PGE modified with MIP and electropolymerized applying different numbers of voltammetric cycles. Polymerization conditions: C_CUR_ = 5.00 × 10^−4^ mol/L; C_DIP_ = 2.50 × 10^−5^ mol/L; supporting electrolyte 0.2 mol/L NaOH; potential scanned between 0.000 and 1.000 V; scan rate 0.100 V/s Figure S4: Comparison of the DIP oxidation signal recorded by DPV in a BRB solution with pH = 3.29 at PGE modified with MIP and electropolymerized at different scan rates (v). Polymerization conditions: C_CUR_ = 5.00 × 10^−4^ mol/L; C_DIP_ = 2.50 × 10^−5^ mol/L; supporting electrolyte 0.2 mol/L NaOH; potential scanned between 0.000 and 1.000 V; 7 voltammetric cycles. Figure S5: Variation of DPV peak current recorded at MIP_PGE for a 1.00 × 10^−6^ mol/L DIP in a BRB solution at pH = 3.29 with the (a) accumulation potential (t_acc_ 30 s) and (b) accumulation time (E_acc_ −0.400 V).
