# Supplementary material for: Curcumin-Based Molecularly Imprinted Polymer Electropolymerized on Single-Use Graphite Electrode for Dipyridamole Analysis
Source: Molecules. 2024 Sep 29;29(19):4630. doi: 10.3390/molecules29194630 (PMC11477586; doi:10.3390/molecules29194630)
Supplement: Supplementary file 1 [file molecules-29-04630-s001.zip › molecules-3186517-supplementary.pdf]

Curcumin Based Molecularly Imprinted Polymer Electropolymerized on Single-Use Graphite Electrode for Dipyridamole Analysis

Daniel Preda [1], Gabriel Lucian Radu [2*], Emilia-Elena Iorgulescu[3], Mihaela-Carmen Cheregi[3], Iulia Gabriela David [3*]

[1] Doctoral School of Chemical Engineering and Biotechnologies, National University of Science and Technology POLITEHNICA Bucharest, Gheorghe Polizu Street 1-7, District 1, 011061, Bucharest, Romania; danielpredaa12@gmail.com (D.P.)

[2] National Institute of Biological Sciences, Centre of Bioanalysis, Splaiul Independentei 296, District 6, 060031, Bucharest, Romania; lucian.radu@incdsb.ro (G.L.R.)

[3] Department of Analytical Chemistry and Physical Chemistry, Faculty of Chemistry, University of Bucharest, Panduri Av. 90-92, District 5, 050663, Bucharest, Romania; emilia-elena.iorgulescu@chimie.unibuc.ro (E.E.I.): mihaela.cheregi@g.unibuc.ro (M.C.C.); gabrielaiulia.david@g.unibuc.ro (I.G.D.)

* Correspondence: lucian.radu@incdsb.ro (G.L.R.); gabrielaiulia.david@g.unibuc.ro (I.G.D.)

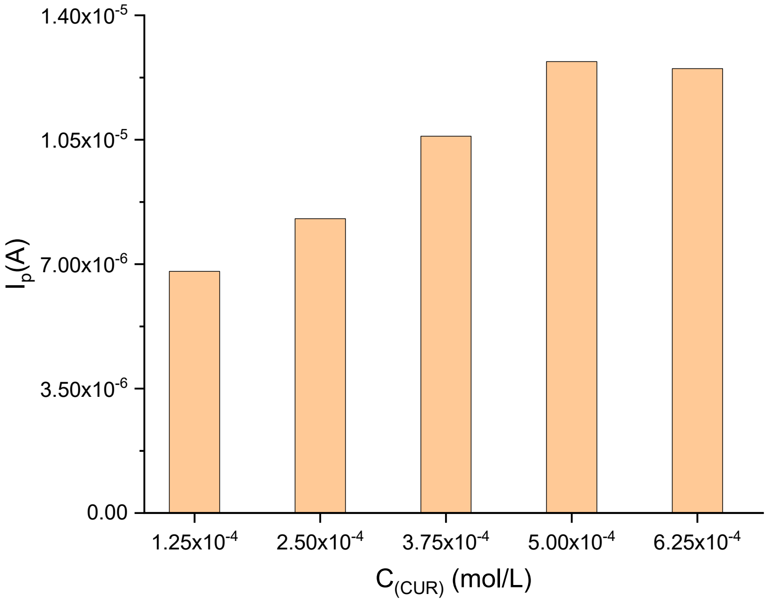

Figure S1. The effect of CUR concentration in the polymerization mixture on the DIP oxidation signal recorded by DPV in BRB solution pH = 3.29 at MIP_PGE. Polymerization conditions: C_{DIP} = 1.00 × 10^{-5} mol/L; supporting electrolyte 0.2 mol/L NaOH; 5 voltammetric cycles between 0.000 and 1.000 V; scan rate 0.100 V/s.

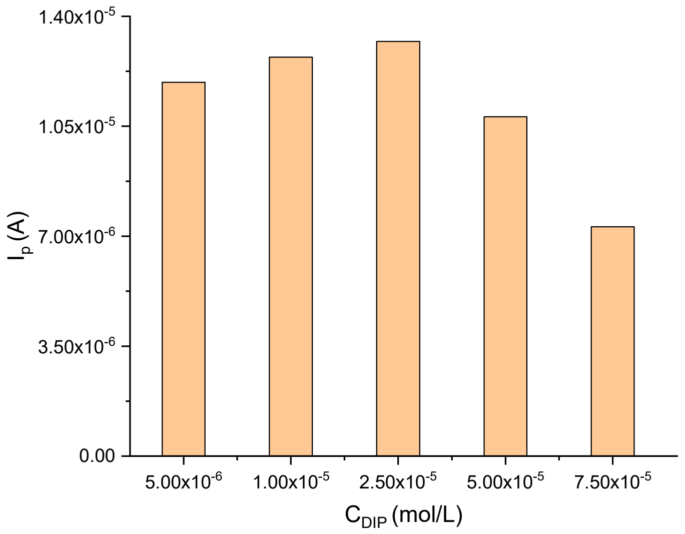

Figure S2. The effect of DIP concentration in the polymerization mixture on the DIP oxidation signal recorded by DPV in BRB solution pH = 3.29 at MIP_PGE. Polymerization conditions: C_{CUR} = 5.00 × 10^{-5} mol/L; supporting electrolyte 0.2 mol/L NaOH; 5 voltammetric cycles between 0.000 and 1.000 V; scan rate 0.100 V/s.

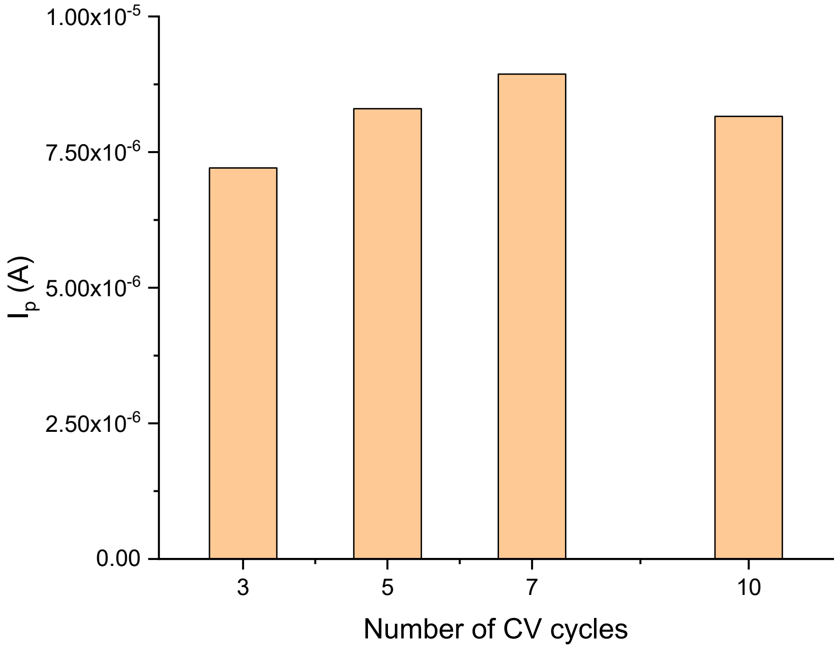

Figure S3. Comparison of the DIP oxidation signal recorded by DPV in BRB solution pH = 3.29 at PGE modified with MIP electropolymerized applying different numbers of voltammetric cycles. Polymerization conditions: C_{CUR} = 5.00 × 10^{-4} mol/L; C_{DIP} = 2.50 × 10^{-5} mol/L; supporting electrolyte 0.2 mol/L NaOH; potential scanned between 0.000 and 1.000 V; scan rate 0.100 V/s.

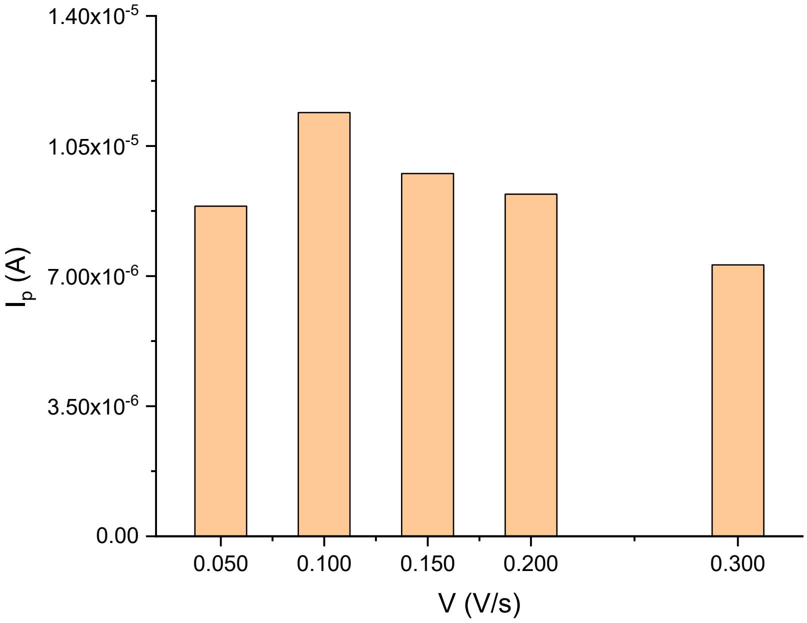

Figure S4. Comparison of the DIP oxidation signal recorded by DPV in BRB solution pH = 3.29 at PGE modified with MIP electropolymerized at different scan rates (v). Polymerization conditions: C_{CUR} = 5.00 × 10^{-4} mol/L; C_{DIP} = 2.50 × 10^{-5} mol/L; supporting electrolyte 0.2 mol/L NaOH; potential scanned between 0.000 and 1.000 V; 7 voltammetric cycles.

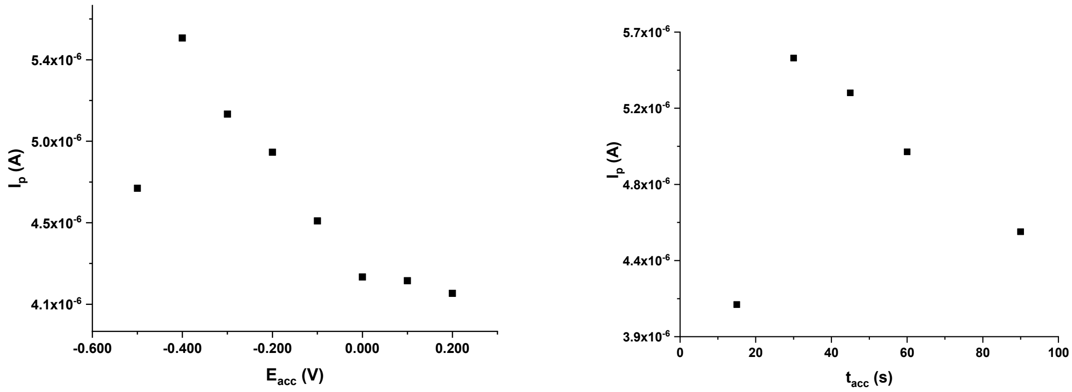

Figure S5. Variation of DPV peak current recorded at MIP_PGE for a 1.00 × 10^{-6} mol/L DIP in BRB solution pH = 3.29 with the (a) accumulation potential (t_{acc} 30 s) and (b) accumulation time (E_{acc} -0.400 V)
